# Modelling count, bounded and skewed continuous outcomes in physical activity research: beyond linear regression models

**DOI:** 10.1186/s12966-023-01460-y

**Published:** 2023-05-05

**Authors:** Muhammad Akram, Ester Cerin, Karen E. Lamb, Simon R. White

**Affiliations:** 1 Mary MacKillop Institute for Health Research, Australian Catholic University, Melbourne, Australia; 2 School of Public Health, The University of Hong Kong, Hong Kong, China; 3 Centre for Epidemiology and Biostatistics, Melbourne School of Population and Global Health, University of Melbourne, Melbourne, Australia; 4 Institute for Physical Activity and Nutrition (IPAN), School of Exercise and Nutrition Sciences, Deakin University, Geelong, Australia; 5 MRC Biostatistics Unit, University of Cambridge, Cambridge, UK; 6 Department of Psychiatry, University of Cambridge, Cambridge, UK

**Keywords:** Count data, Skewed data, Bounded data, Physical activity, Linear regression model, Generalized linear model, Transformations

## Abstract

**Background:**

Inference using standard linear regression models (LMs) relies on assumptions that are rarely satisfied in practice. Substantial departures, if not addressed, have serious impacts on any inference and conclusions; potentially rendering them invalid and misleading. Count, bounded and skewed outcomes, common in physical activity research, can substantially violate LM assumptions. A common approach to handle these is to transform the outcome and apply a LM. However, a transformation may not suffice.

**Methods:**

In this paper, we introduce the generalized linear model (GLM), a generalization of the LM, as an approach for the appropriate modelling of count and non-normally distributed (i.e., bounded and skewed) outcomes. Using data from a study of physical activity among older adults, we demonstrate appropriate methods to analyse count, bounded and skewed outcomes.

**Results:**

We show how fitting an LM when inappropriate, especially for the type of outcomes commonly encountered in physical activity research, substantially impacts the analysis, inference, and conclusions compared to a GLM.

**Conclusions:**

GLMs which more appropriately model non-normally distributed response variables should be considered as more suitable approaches for managing count, bounded and skewed outcomes rather than simply relying on transformations. We recommend that physical activity researchers add the GLM to their statistical toolboxes and become aware of situations when GLMs are a better method than traditional approaches for modeling count, bounded and skewed outcomes.

**Supplementary Information:**

The online version contains supplementary material available at 10.1186/s12966-023-01460-y.

## Background

In the field of physical activity, outcome (or response) variables are often bounded (typically, taking only non-negative values) and positively skewed [1–3]. For example, studies examining minutes of leisure time physical activity in the past week (i.e., a non-negative variable) could include a substantial number of people reporting less than 30 weekly minutes of this specific physical activity domain, few reporting 30 to 59 min, and even fewer reporting at least 60 min (i.e., a skewed distribution).

Physical activity is challenging to measure accurately. Data on physical activity have been collected in a variety of ways including self-report questionnaires, such as the International Physical Activity Questionnaire (IPAQ) [4], and objective measurements, such as accelerometers [5], pedometers [6] and combinations of heart rate monitors and movement sensors [7]. These methods allow estimation of a diverse range of physical activity outcomes, such as the number of days during which participants engaged in walking for transport in the last or usual week [8, 9], daily or weekly minutes spent in moderate-to-vigorous physical activity (MVPA) [10], average number of daily steps [11] or mean accelerometer counts per minute [12]. Non-normally distributed outcomes are prevalent in cross-sectional [10, 13], longitudinal [1, 2] and intervention studies [3, 14] of physical activity. Often, physical activity researchers are interested in examining associations of socio-demographic characteristics, such as gender, age, education [15], or environmental [10] and psychosocial factors [16] with physical activity outcomes. Many researchers apply linear regression models (LMs) or related (e.g., analysis of variance (ANOVA)) methods [17–22]. However, these methods rely on assumptions about the underlying distribution of the data (e.g., model residuals are assumed to be normally distributed) which may not be appropriate when modelling physical activity outcomes.

If skewed and bounded physical activity outcomes are not modelled appropriately there may be issues with the validity of interpretations and conclusions. If the outcome is a count (non-negative integer [i.e., 0, 1, 2, …]), LMs are likely to be inappropriate because these models will generate non-integer predicted values and may generate negative predicted values – which are impossible – as LMs assume the outcome can take any real number. For example, assuming few individuals are active every day, the number of days per week of vigorous physical activity (a discrete variable), could be modelled as count data. Continuous bounded and skewed data in physical activity research include minutes of leisure-time physical activity or accelerometer-based MVPA per week. These are bounded as there are lower (i.e., 0 min) and upper limits (i.e., 10,080 min corresponding to the maximum possible minutes of PA per week) for minutes of the week and distributions are often skewed. These outcomes can be modelled using readily available bounded and skewed distributions.

In practice, when handling count, bounded and skewed outcomes several different approaches have been used. These include ignoring the distributional issues and conducting the analyses on the original outcome, applying some type of transformation (for example the Box-Cox transformation [23]) to obtain an approximately normally distributed outcome variable, or applying regression models that are appropriate for the type of outcome (for example GLMs) [24, 25]. However, deciding what is the best approach, or what is “good enough”, can be tricky. The aim of this paper is to discuss regression-based approaches for dealing with count, bounded and skewed outcome data and to demonstrate the use of these methods as applied to the Active Lifestyle and Environment in Chinese Seniors (ALECS) study of Hong Kong older adults that collected discrete as well as continuous physical activity data [26, 27]. The focus of this article is on physical activity as the outcome variable.

## Methods

In the following sub-sections, we discuss the various analytical approaches that deal with count, bounded and skewed continuous outcome data. These approaches employ more appropriate probability models to handle count, bounded and skewed outcomes, rather than trying to make things fit into the usual (and inappropriate) normal-based LMs.

### Count data

As mentioned previously, physical activity data are often discrete (i.e., can take a finite or countable number of possible values); count data are a special case taking non-negative integers (i.e., 0, 1, 2, …). For example, the self-reported number of days per week a person engages in at least 20 min of vigorous physical activity is discrete, taking the values 0 to 7. However, this variable could be considered as a count if the outcome is skewed towards lower values (meaning a count model is unlikely to generate invalid values greater than the upper bound of 7). GLMs, such as the Poisson regression model or one of its variants (e.g., negative binomial regression), are appropriate and well-suited to modelling count data [28]; providing valid inference for such variables in terms of estimates, *p*-values and confidence intervals (CIs).

#### Poisson regression model

The simplest distribution commonly used for modelling count data is the *Poisson distribution*. A Poisson regression model is similar to a LM, with two exceptions. Firstly, it assumes that the response follows a Poisson distribution. Secondly, rather than modelling the expected value of the response as a linear function of the regression coefficients, it includes a link function that relates the expected value of the response to the linear function of the coefficients, where the link function in Poisson regression is the natural logarithm (see [28, 29] for details).

The Poisson model is useful for describing the mean of an outcome variable (conditional on values of the covariates) and assumes that the mean and variance are equal. Although Poisson regression is fundamental to the regression analysis of count data, it is often of limited utility for real data due to the assumption that the mean and variance of the outcome distribution are equal. In many practical applications, count data exhibit far greater variability than is predicted by the Poisson distribution, a phenomenon known as over-dispersion (i.e., the variance is larger than mean). Although under-dispersion (i.e., the variance is smaller than the mean) is also possible, it is far less common. Neglecting over-dispersion in regression models for count data results in the standard errors of the parameter estimates being underestimated which consequently affects the CIs and *p*-values. Thus, accounting for over-dispersion when modelling count data is essential. Failing to deal with these features of the data can lead to biased standard errors of parameter estimates and, thus, invalid conclusions.

#### Quasi-Poisson and negative binomial regression models

To deal with over-dispersion, there are two common extensions of the Poisson model. The first is a quasi-Poisson model that includes an extra parameter which estimates how much larger the variance is than the mean. This parameter estimate (also known as the dispersion parameter) is then used to correct for the effects of the larger variance on the standard errors, CIs and *p*-values. Note that quasi-Poisson GLMs reduce to Poisson GLMs when this dispersion parameter is equal to one. The second is a negative binomial model. Like Poisson regression, negative binomial regression is suitable for modelling count outcome data but is more flexible as it has an additional parameter to account for over-dispersion.

A natural question for researchers in the physical activity field is: which model should be used in the presence of over-dispersion? There is no general recommendation as to which of the two options is best, making it difficult to specify *a priori* which of the quasi-Poisson and negative binomial models are most suitable. However, in practice, there is typically no great difference when comparing these models [30]. Therefore, either approach should be suitable if over-dispersion is of concern, although the best fitting model can be chosen by fitting both regression models and comparing the models using an information criterion value, such as the Akaike Information Criterion (AIC).

#### Example 1

We illustrate the LM, GLM-Poisson, GLM-quasi-Poisson, and GLM-Negative Binomial described above by applying them to data from ALECS study, using the weekly minutes of self-reported physical activity (in a usual week) among 402 adults (62 years and older, mean (sd) age 75.6 (6.2) years; 69% female) from Hong Kong. In this example, the outcome variable is the self-reported habitual physical activity (PA) expressed as weekly minutes, and the number of chronic medical conditions (NCMC) is the exposure of interest. We note that weekly minutes is strictly a discrete outcome. However, we can safely treat it as a count outcome as we are unlikely to be affected by the upper bound described earlier. All models adjust for age and sex, characteristics commonly prognostic of PA.

### Bounded and skewed continuous data

The LM approach is appropriate for continuous normally distributed data. For non-normally distributed continuous data, LMs may still be appropriate in studies with a large sample size [31] where we are more interested in the mean outcome rather than accurate and powerful methods of inference. However, there are many other pitfalls that may affect the quality of the interpretation and conclusions drawn from poorly fitted models [32]. Moreover, variables that take zero as a minimum value (such as minutes of leisure time PA in the past week), namely bounded outcomes, can prove problematic to model using LMs as this approach assumes that the outcome variable can take both negative and positive values. Therefore, there is a potential for predicted values from a LM to take impossible values. Alternate approaches, such as the analysis of data on a transformed scale that yields an approximately normal distribution, may provide a better representation of the way in which the outcome variable takes on values. Furthermore, models that are specifically designed to take skewness into account often perform much better than LMs using transformations. In this section we aim to provide appropriate alternative modelling strategies for skewed bounded continuous data.

#### Transformation bias and impact on interpretation

When the distribution of a continuous outcome is non-normal, transformations (e.g., Box-Cox transformation [23]) of data can be applied to make data as normal as possible. The log transformation is, arguably, the most popular among the different types of transformations due to its ease of use and interpretability. Another commonly chosen transformation is the square root of the outcome. This is sometimes used by researchers instead of the log transformation when the outcome can take values of zero; since the log of zero is undefined. However, there is no guarantee that the transformation chosen will reduce skewness and make the data a better approximation of the normal distribution. In fact, in some cases applying the transformation can make the distribution more skewed than the original data [33]. Moreover, data transformations are somewhat unsatisfactory as the resulting models no longer pertain directly to the original scale of measurement, which is usually of greatest interest [34]. Transformations try to force a model framework and distributional assumption that may not be best for the data. For example, the back-transformation of square root transformed outcome or natural log outcomes can make the interpretation of findings challenging [35]. Furthermore, statistically significant differences on the transformed scale are uninformative as to whether significant differences exist on the original untransformed scale and vice versa [36].

A key issue with the use of transformations is that transformations can induce a transformation bias. The predictions of the true population mean will be biased (over or under estimated) because the regression models the transformed outcome (i.e., $E\left[{log}\left(y\right)\right]=\alpha +x\beta$) rather than the transformed expected value (i.e., ${log}\left[E\left(y\right)\right]=\alpha +x\beta$) [37]. For example, the mean of the log-transformed observations, $E\left[log\left(y\right)\right]$, is often used to estimate the population mean of the original data by applying the anti-log (i.e., exponential) function to obtain ${exp}\left[E\left[log\left(y\right)\right]\right]$. However, this inversion of the mean log value does not usually result in an appropriate estimate of the mean of the original data. Many researchers tend to interpret ${exp}\left[E\left[log\left(y\right)\right]\right]$ as the mean of *y*. However, ${exp}\left[E\left[log\left(y\right)\right]\right]$ and $E\left(y\right)$ are quite different quantities (see [33] for details).

Interpreting regression coefficients typically involves determining what change in the dependent variable is suggested by a given change in each independent variable. However, we cannot back transform coefficient estimates or interval estimates of coefficients of transformed outcomes, for example the LM of the square-root transformed outcome (sqrt-LM), because the simple back (reverse) transformation does not necessarily map back onto the original (untransformed) effect of interest [37, 38]. In fact, the interpretation of the regression coefficient from sqrt-LM is much more complex, as described by Huber [39]. The regression coefficients, ${b}_{i}$, from sqrt-LM indicate that a one unit change in exposure (${X}_{i}$) is associated with a $2{b}_{i}\sqrt{Y}$ change in the outcome.

#### Gamma and inverse gaussian regression

The binomial (for binary outcomes), Poisson (count outcomes) and Gaussian (normal outcomes) GLMs are commonly used, but there are other GLMs for other particular types of outcome [28]. For example, the gamma and inverse Gaussian are intended for positive, continuous, skewed responses. Many applications have response variables which are continuous and positive (such as minutes of leisure time PA in the past week).

A gamma GLM is appropriate in situations where the variance increases linearly with the mean response [40]. The practical uses of gamma have been discussed in de Jong and Heller [41], Frees [42] and Venables and Ripley [43].

The inverse Gaussian distribution, similar to that of the gamma, with a high initial peak, rapid drop-off and long right tail, has been found to be an appropriate reflection of right skewed positive data, such as insurance claims and length of hospital stay [44]. The inverse Gaussian regression is also suitable for data that are continuous, non-negative, and right skewed. The variance function for the inverse Gaussian GLM increases more rapidly with the mean (variance = mean^3^) than the gamma GLM (variance = mean^2^), making it suitable for data where this occurs (see [28] and [40] for details).

#### Example 2

We illustrate the regression models for skewed continuous data described above by applying them to data from the ALECS study, using daily average minutes (i.e., a continuous outcome) of MVPA (mean = 25.7; sd = 23.3) as our outcome and same covariates as used in Example 1, i.e., age, sex and NCMC.

## Results

**Fig. 1:**
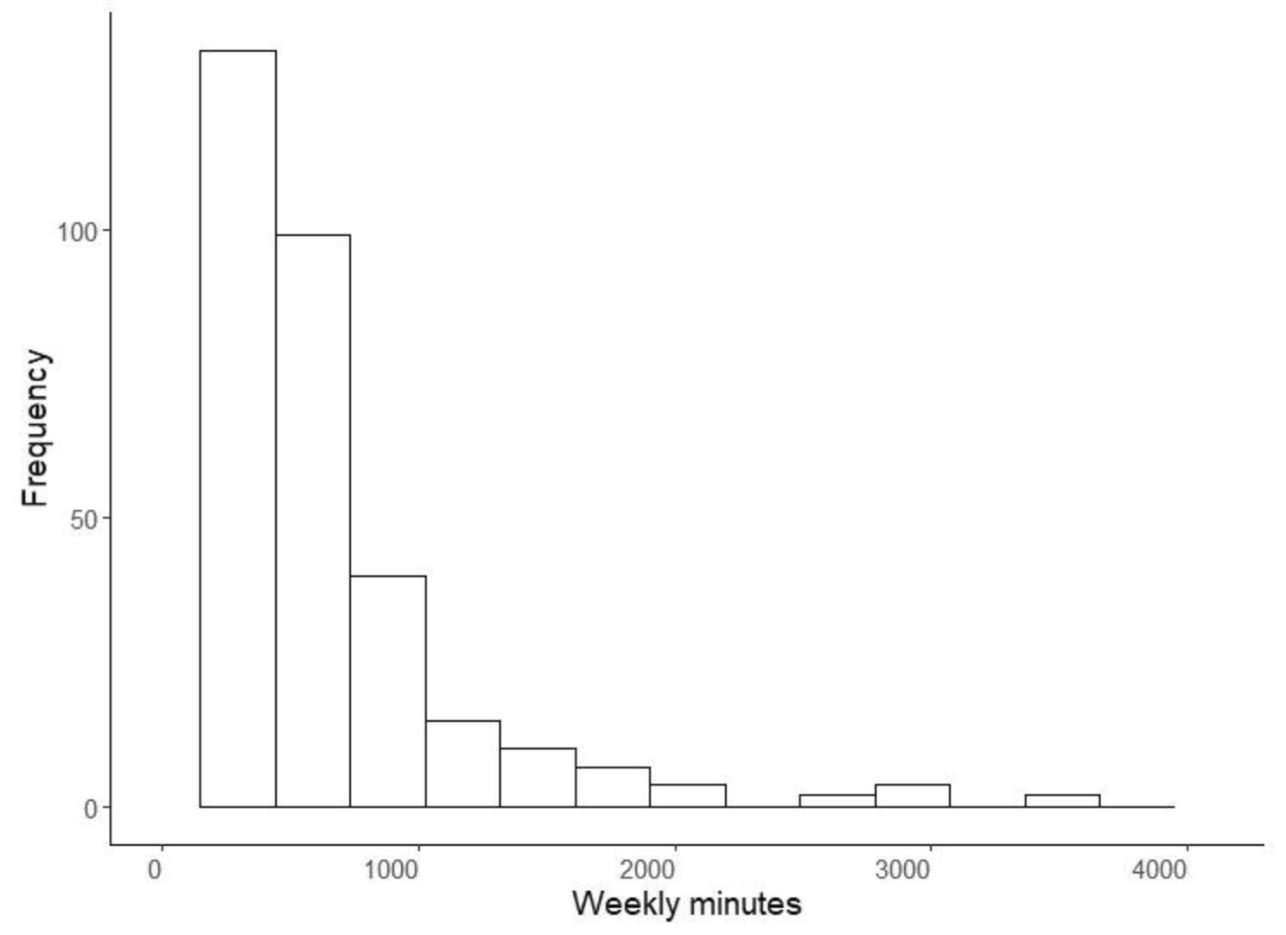
Distribution of weekly minutes of habitual physical activity from the ALECS study of physical activity in Hong Kong older adults (n = 402)

The descriptive measures of the variables used in this study are presented in Table 1. The assumption that the variance is equal to the mean (required for Poisson regression) may not be valid for the PA outcome (third row) and Fig. 1 clearly indicates that the outcome is skewed.

**Table 1:** Descriptive statistics for selected variables from the ALECS study of physical activity in Hong Kong older adults (N = 402)

Variable	Minimum	Q_1_	Median	Q_3_	Maximum	Mean	Variance
**Age (years)**	62.9	70.8	75.2	80.0	97.9	75.6	37.8
**NCMC**	0	2	3	5	10	3.1	4.1
**PA (min/week)**	0	200	390	670	4074	537.4	322,650

PA: Physical activity; NCMC: Number of chronic medical conditions; Q_1_: first quartile; Q_3_: third quartile

### LM vs. regression models for count data – results of example 1

The LM and different regression models for count data fitted to the PA outcome are presented in Table 2. The results are very similar (in terms of statistical significance) for all models, except for the Poisson regression model, indicating both age and NCMC are not associated with PA. In contrast, the Poisson regression indicates a significant association between both age and NCMC with PA. From Table 2, it can also be noticed that the estimated coefficients are very similar across the models, except for the LM. This is because the LM produces estimates on the original measurement unit of the outcome variable (weekly minutes of PA) rather than on a link (i.e., natural logarithmic) scale as the GLMs considered do (the interpretation of the coefficients is explained later in the paper). However, the standard errors (and 95% CI) differ. For example, if we consider the exposure variable NCMC, we can see that the standard error from the Poisson regression model is much smaller than those of the other GLMs, indicating possible over-dispersion which we anticipated would be present from examination of the descriptive statistics since the variance of the PA outcome was much larger than the mean (Table 1). In Poisson regression, over-dispersion will result in an underestimation of the standard errors of the estimated regression coefficients because these errors reflect only the variation expected from a Poisson distribution.

**Table 2:** Estimated associations between PA and NCMC adjusted for age and sex from the ALECS study of physical activity in Hong Kong older adults using various regression models (N = 402)

	LM	GLM-Poisson	GLM-quasi-Poisson	GLM-Negative Binomial
**Intercept**				
Estimate	875.5	6.918	6.918	7.021
SE	349.1	0.027	0.645	0.624
p-value	0.013	< 0.001	< 0.001	< 0.001
95% CI	189.2, 1562	6.87, 6.97	5.66, 8.19	5.77, 8.25
**Age (years)**				
Estimate	-3.997	-0.007	-0.007	-0.009
SE	4.668	0.0004	0.009	0.008
p-value	0.392	< 0.001	0.388	0.305
95% CI	-13.17, 5.18	-0.008, -0.007	-0.025, 0.009	-0.025, 0.008
**Sex** (Female reference)				
Estimate	82.320	0.151	0.151	0.149
SE	61.365	0.005	0.111	0.110
p-value	0.181	< 0.001	0.173	0.175
95% CI	-38.32, 203.0	0.14, 0.16	-0.07, 0.37	-0.06, 0.37
**NCMC**				
Estimate	-20.078	-0.038	-0.038	-0.046
SE	14.180	0.001	0.027	0.025
p-value	0.158	< 0.001	0.153	0.072
95% CI	-47.95, 7.80	-0.041, -0.036	-0.091, 0.014	-0.099, 0.009
**AIC**	6244.3	183,071	5873.9	5863.4
**DP**	-	-	586.3	0.976

SE: standard error; CI: confidence interval; LM: linear regression model; NCMC: number of chronic medical conditions; AIC: Akaike information criterion; DP: dispersion parameter. All analyses were conducted in R version 4.0.2 [45]

To identify the best fitting model, the AIC [24] value can be used to compare the models. The preferred model is the one with the minimum AIC value, although the appropriateness of the model for the outcome under consideration must also be taken into account. Though an inspection of *p*-values suggests that the conclusions from various models presented in Table 2 seem very similar (except those based on the Poisson regression), a comparison of the AIC values of these models reveals that the quasi-Poisson and negative binomial regression models are superior to both the Poisson regression and the LM; the negative binomial performs the best with the lowest AIC. As mentioned, the Poisson regression is inferior because there is substantial over-dispersion in the Poisson regression model and, consequently, the standard errors of the Poisson regression are underestimated. Tests to assess over-dispersion can be conducted. The dispersion parameter was assessed for both the quasi-Poisson regression and negative binomial regression (see DP in Table 2). The estimate of the dispersion parameter from the quasi-Poisson regression was greater than one (*p* < 0.001), while an estimate greater than zero (*p* < 0.001) was obtained for the negative binomial regression [25, 46]. Note that the LM appears superior to the Poisson regression based on the AIC. However, the LM is a poor choice as it is inappropriate for modelling count data. Therefore, it is important to be aware that the AIC alone is not sufficient for selecting an appropriate model. Specifically, there are important aspects of the model checking that AIC would miss.

#### Interpreting GLMs (compared to LMs)

The GLMs above produce coefficient estimates on a natural logarithmic scale. Taking the exponential of the coefficients for Poisson and negative binomial regression models provides incidence rate ratios (IRRs), which indicate the proportional difference in outcome associated with a unit difference in exposure variable. IRRs of 1 indicate no difference in the outcome by exposure; IRRs greater than 1 indicate that the outcome increases as the exposure increases and IRRs less than 1 indicate a decrease in outcome as the exposure increases. For example, consider the coefficient of NCMC in the Poisson regression model. To obtain the IRR, the coefficient should be exponentiated, 0.963 $\left({e}^{-0.038}\right)$. As the IRR is below 1, this suggests that the outcome decreases for each unit increase in the NCMC (i.e., for each additional NCMC). A more meaningful way of expressing this is to present the percentage decrease in the outcome for each additional NCMC; estimated to be 3.7% for this coefficient $\left((1-{e}^{-0.038}\right)\times 100=3.7\%)$. In other words, PA decreases by 3.7% with each additional NCMC. Importantly, the IRR should be interpreted along with the 95% CI. The 95% CI for the NCMC coefficient ranges from − 0.041 to -0.036 which exponentiated results in a 95% CI of 0.960 to 0.965 for the IRR. This suggests a decrease in PA ranging from 3.5% $(\left(1-{e}^{-0.036}\right)\times 100)$ to 4.0% $(\left(1-{e}^{-0.041}\right)\times 100)$ with each additional NCMC. The interpretation of coefficients for the negative binomial and quasi-Poisson regression models were consistent with the Poisson regression model. However, the exponentiated 95% CIs contain 1 for each of these models meaning it is possible that there is no change in PA with each additional NCMC. Since we found over-dispersion in the data, the results from either the quasi-Poisson or negative binomial regression are more appropriate (i.e., there is insufficient evidence that age and NCMC are associated with PA). Note that it is common to present the IRR and corresponding 95% CI for the IRR in results tables for these GLMs rather than the coefficients in the log scale as shown in Table 2.

### LM vs. regression models for continuous positively-skewed data – results of example 2

**Fig. 2:**
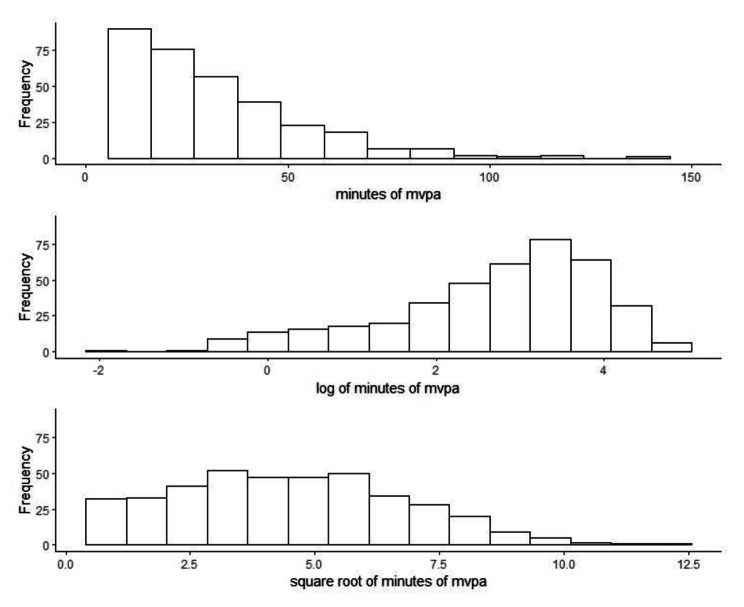
Distribution of the original and transformed daily average minutes of MVPA for older adults from the ALECS study of physical activity in Hong Kong (n = 402)

Figure 2 shows the distributions of the original, untransformed MVPA data as well as the transformed data (employing the commonly used log and square root transformations). Whilst we can clearly see that daily average minutes of MVPA are, as expected, positively skewed (Fig. 2; top panel), the transformed data do not represent an obviously better approximation to the normal distribution (bottom two panels in Fig. 2). In fact, the transformed data appear to be substantially negatively skewed (log-transform) and bounded (square root-transform).

A comparison of fitted models is presented in Table 3. All models yielded similar results in terms of statistical significance. There is strong evidence that age is negatively associated with MVPA (i.e., older participants engage in fewer minutes of MVPA) and that, on average, males engage in more minutes of MVPA than females.

Although an inspection of the *p*-values suggests that the conclusions from the various models presented in Table 3 are very similar, we can use the AIC values to assist in identifying the best fitting model, as in Example 1. Here we need to note that the AIC values of transformed outcome models (log or square root) are not comparable with those of non-transformed outcome models because they are on different outcome data. Therefore, to enable model comparison, the AIC values from the models of transformed MVPA data need to be adjusted following a procedure described by Akaike [24](page 224). Table 3, thus, presents adjusted AIC values for the transformed MVPA models. The AICs suggest that the performance of the gamma regression model (GLM-gamma) and LM of the square-root transformed outcome (sqrt-LM) are very similar and the best among all the fitted models. In choosing between GLM-gamma and sqrt-LM, it should be remembered that the transformation can make it tricky to interpret the findings on the original scale. For example, when researchers are interested in how the average PA outcome differs between groups, or increases with each unit increase of a covariate, the back-transformation of coefficients does not provide these estimates and, as a consequence, can make the interpretation of findings challenging. On the other hand, the GLM-gamma not only performs slightly better than sqrt-LM in terms of AIC values but also avoids transformation bias, and is suitable for data that are continuous, non-negative and right (positively) skewed. As discussed in Example 1, AIC values alone are not sufficient to choose an appropriate model. Considering the continuous outcome, model selection should also be based on diagnostic plots of model fit (see Appendix A).

**Table 3:** Estimated associations of age, sex and NCMC with MVPA from different regression models for the ALECS study of physical activity in Hong Kong older adults (n = 402)

	LM	sqrt-LM	log-LM	GLM-gamma	GLM-IG
**Intercept**					
Estimate	115.567	14.835	8.754	7.742	8.548
SE	12.438	1.202	0.673	0.521	0.537
p-value	< 0.001	< 0.001	< 0.001	< 0.001	< 0.001
95% CI	91.10, 140.00	12.47, 17.20	7.43, 10.08	6.66, 8.81	7.39, 9.69
**Age (years)**					
Estimate	-1.263	-0.145	-0.085	-0.065	-0.076
SE	0.166	0.016	0.009	0.007	0.007
p-value	< 0.001	< 0.001	< 0.001	< 0.001	< 0.001
95% CI	-1.59, -0.94	-0.18, -0.11	-0.10, -0.07	-0.08, -0.05	-0.09, -0.06
**Sex** (Female reference)					
Estimate	19.593	1.885	0.899	0.754	0.816
SE	2.186	0.211	0.118	0.092	0.123
p-value	< 0.001	< 0.001	< 0.001	< 0.001	< 0.001
95% CI	15.29, 23.89	1.47, 2.30	0.67, 1.13	0.58, 0.94	0.59, 1.08
**NCMC**					
Estimate	-0.177	0.010	0.029	0.012	0.027
SE	0.505	0.049	0.027	0.021	0.022
p-value	0.727	0.841	0.289	0.559	0.219
95% CI	-1.17, 0.82	-0.09, 0.11	-0.02, 0.08	-0.03, 0.05	-0.01, 0.07
**Adjusted-AIC**	3563	3321	3377	3320	3599

LM: linear regression model fit on untransformed MVPA; sqrt-LM: linear regression model fit on square-root transformed MVPA; log-LM: linear regression model fit on log transformed MVPA; GLM: generalized linear model; IG: inverse Gaussian; SE: standard error; CI: confidence interval; AIC: Akaike Information Criterion

**Fig. 3:**
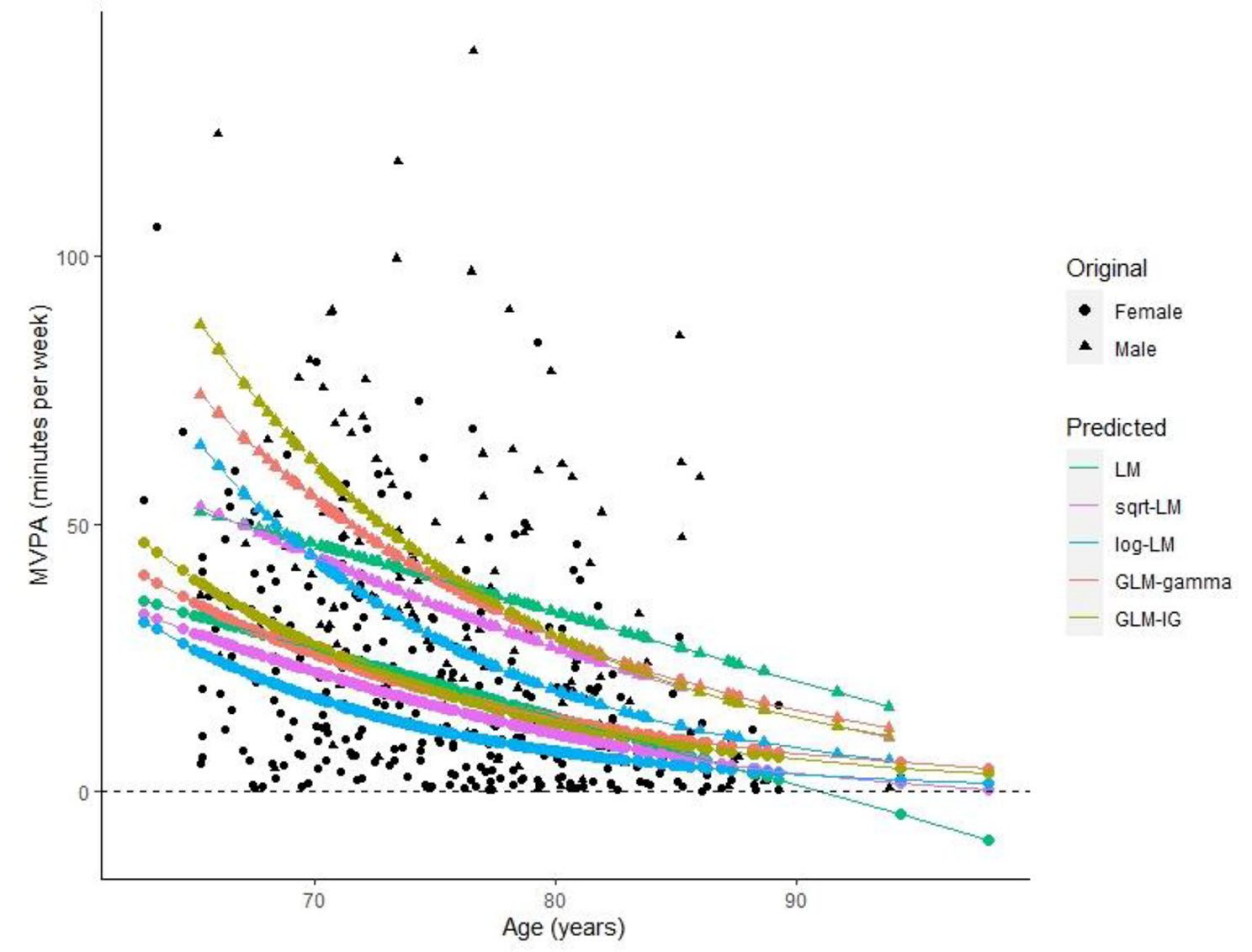
Observed and predicted values from various regression models of daily average minutes of MVPA for the ALECS study of physical activity in Hong Kong older adults

Furthermore, the predicted values of MVPA from all the fitted models along with observed MVPA are also plotted in Fig. 3 against age, and at fixed values of NCMC = 0. It can be seen from this figure that the predicted values of MVPA from each fitted model show two separate lines (sets of points), one for male and one for female (since the sex coefficient is significant, a sex-difference is expected). It can be clearly seen from this plot that the untransformed LM produced some negative predicted values of the mean daily minutes of MVPA, which are impossible values for the response variable. This shows that the LM is not appropriate when the outcome is skewed, bounded and non-negative.

#### Interpreting sqrt_LM (compared to GLM)

As discussed in the transformation section above, interpreting coefficients under transformations is non-trivial. The age coefficient in sqrt-LM indicates that every year increase in age, after accounting for sex and NCMC, is associated with a change in the daily average minutes of MVPA of 2${b}_{i}$ = 2 × (-0.145) = -0.290 times the square root of the current daily average minutes of MVPA. It can be more illustrative to consider examples of how the outcome will change for possible values of the exposure. For example, for a person with an average of 60 min of MVPA, then a one-year increase in age of that person is associated with a decrease of 2.24 (-0.290 × $\sqrt{60}$ ) daily average minutes of MVPA (negative coefficient estimate indicates a decrease). The 95% CI for the age coefficient ranges from − 0.18 to -0.11

which suggests a decrease of 1.70 to 2.79 daily average minutes of MVPA. In comparison, from the GLM-gamma model, for every year increase in age (after adjusting for sex and NCMC) the daily average minutes of MVPA would be expected to decrease by a factor of $\left({e}^{-0.065}\right)=0.937$ (or expected to decrease by $\left(1-0.937\right)\times 100=6.3\%)$. Put simply, each year increase in age is associated with a decrease of 6.3% in the daily average minutes of MVPA. The 95% CI can be interpreted in similar way as described in Example 1. Note that, as mentioned in Example 1, it is usual to present the IRR and corresponding 95% for the IRR in results tables for GLMs rather than the coefficients on the log scale as shown in Table 3.

## Discussion

Physical activity researchers have often relied on LMs when assessing the relationship between predictor(s) and a PA outcome (count/continuous), even when the assumptions of the traditional model (e.g., normal distribution) are not satisfied. In practice, when handling skewed outcomes, most apply some type of transformation to obtain a normally distributed outcome variable [17–22]. However, transformations do not always normalize distributions. Further, the choice of transformation can make it tricky to interpret the findings on the original scale. For example, when researchers are interested in how the average PA outcome differs between groups (e.g., sex), or increases with each unit increase of a covariate (e.g., age), the back-transformation of coefficients does not provide these estimates, and consequently, can make the interpretation of findings challenging.

GLMs offer an alternative to the LM, especially for count, bounded and skewed outcomes commonly encountered in PA research. GLMs allow for response variables that have non-normal distributions. Although these GLMs offer potential, care has to be taken as it is still possible that bounded outcomes (for example, the number of days a week doing PA is bounded above by 7) could have observations outside the plausible range using Poisson (although, we would not get negative predictions). Furthermore, it is important to be aware that the AIC alone is not sufficient for selecting an appropriate model. While AIC can be helpful to guide model selection, it is important to reflect on what is the most appropriate modelling choice for the type of data being considered.

This tutorial paper provides an introductory overview of appropriate methods for handling count, bounded and skewed continuous outcome data. However, alternative approaches may be required when there are a large proportion of zeros values in the count or skewed continuous outcome variable, i.e., when there are more zeros than expected under a standard count/continuous model. Conventional distributions usually cannot effectively explain and model this type of data. For this reason, different models which can account for a large proportion of zero observations must be applied instead [47]. Furthermore, the models presented assume all observations are independent. Correlated data arise frequently in PA research (e.g., longitudinal studies, clustered data) [48, 49]. Ignoring correlation in regression analysis can lead to incorrect conclusions, and therefore regression models that account for correlation should be applied instead [48, 49]. Finally, although we discuss common approaches for handling skewed data, other methods exist which are outside the scope of this paper, such as bootstrapping [50], the skew-t distribution [51–53], as well as non-parametric and semi-parametric approaches [54].

## Conclusion

GLMs which more appropriately model non-normally distributed response variables should be considered as more suitable approaches for managing count, bounded and skewed outcomes rather than simply relying on transformations. Overall, GLMs can be a valuable alternative to LM. We recommend that PA researchers add the GLMs to their statistical toolboxes and become aware of situations when GLMs might be a better method than traditional approaches for modelling count, bounded and skewed continuous outcomes.

## Electronic supplementary material

Below is the link to the electronic supplementary material.

Supplementary Material 1: A supplementary file with examples of R script for all models that have been fitted in this paper.

Supplementary Material 2: A supplementary file with examples of STATA script for all models that have been fitted in this paper.

Supplementary Material 3: A supplementary file with examples of SAS script for all models that have been fitted in this paper.

Supplementary Material 4: A supplementary file with examples of SPSS script for all models that have been fitted in this paper.

Supplementary Material 5: Appendix A: comparison between GLM-gamma and GLM-IG models.

## Data Availability

The data used in this tutorial are available from Ester Cerin (Ester.Cerin@acu.edu.au) upon reasonable request.

## Abbreviations

PA: physical activity
MVPA: moderate-to-vigorous physical activity
ALECS: the Active Lifestyle and Environment in Chinese Seniors
LM: Linear regression model
sqrt-LM: linear regression model fit on square-root transformed data
log-LM: linear regression model fit on log transformed data
GLM: generalized linear model
GLM-gamma: generalized linear model with gamma distribution
GLM-IG: generalized linear model with inverse Gaussian distribution
NCMC: number of chronic medical conditions
SGML: ML, maximum likelihood.
SE: standard error
CI: confidence interval
NB: negative binomial.
AIC: Akaike information criteria
