# Supplementary material for: Modelling count, bounded and skewed continuous outcomes in physical activity research: beyond linear regression models
Source: Int J Behav Nutr Phys Act. 2023 May 5;20:57. doi: 10.1186/s12966-023-01460-y (PMC10163772; doi:10.1186/s12966-023-01460-y)
Supplement: Supplementary file 5 — Supplementary Material 5: Appendix A: comparison between GLM-gamma and GLM-IG models. [file 12966_2023_1460_MOESM5_ESM.docx]

**Appendix A**

To further illustrate the comparison between GLM-gamma and GLM-IG, the deviance residuals were plotted against the predicted values from these models in Figure 4 (following Faraway[42]). The deviance residuals represent the contributions of individual cases to the deviance. More specifically, they are defined as the signed square roots of the unit deviances [26]. Thus, the deviance residuals are analogous to the conventional residuals. It can be seen from Figure 4 that the plot of residuals versus predicted values obtained from GLM-IG shows a decreasing spread of residuals for larger predicted values, indicating that the variance is not constant and is decreasing as the mean increases. This indicates that the inverse Gaussian variance function is not optimal for these data (that is, the hypothesized mean-variance relationship [variance = mean^3^] for the inverse-Gaussian distribution was too extreme), suggesting that the model is not adequate [42]. On the other hand, the plot of residuals versus predicted values obtained from the GLM-gamma does not exhibit any particular pattern, the residuals are random around zero indicating that the variance is near constant. This once again shows that GLM-gamma is a better choice compared to GLM-IG.


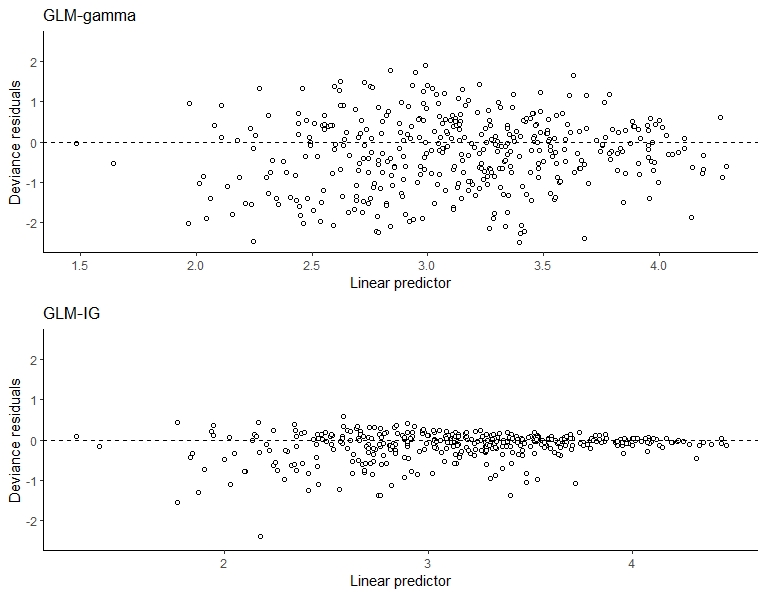


**Figure 4: Deviance residuals versus predicted values plots from GLM-gamma and GLM-IG models of daily average minutes of MVPA for the ALECS study of physical activity in Hong Kong older adults**
